# Double-stranded RNA released from damaged articular chondrocytes promotes cartilage degeneration via Toll-like receptor 3-interleukin-33 pathway

**DOI:** 10.1038/cddis.2017.534

**Published:** 2017-11-02

**Authors:** Changwei Li, Kaizhe Chen, Hui Kang, Yufei Yan, Kewei Liu, Changjun Guo, Jin Qi, Kai Yang, Fei Wang, Lei Guo, Chuan He, Lianfu Deng

**Affiliations:** 1 Shanghai Key Laboratory for Prevention and Treatment of Bone and Joint Diseases with Integrated Chinese-Western Medicine, Shanghai Institute of Traumatology and Orthopedics, Ruijin Hospital, Shanghai Jiaotong University School of Medicine, 197 Ruijin 2nd Road, Shanghai 200025, People's Republic of China; 2 Department of Orthopedics, Ruijin Hospital, Shanghai Jiaotong University School of Medicine, 197 Ruijin 2nd Road, Shanghai 200025, People's Republic of China; 3 Department of Orthopedics, Shanghai Tenth People's Hospital, Tongji University School of Medicine, Shanghai 200072, People's Republic of China

## Abstract

Pattern recognition receptors (PRRs), including Toll-like receptor 3 (TLR3), are involved in arthritic responses; however, whether interleukin-33 (IL-33) is involved in TLR3-mediated cartilage degeneration is unknown. Here, we found that IL-33 was abundantly increased in chondrocytes of osteoarthritis, especially the chondrocytes of weight-bearing cartilage. Furthermore, double-stranded RNA (dsRNA) released from damaged articular chondrocytes induced by mechanical stretching upregulated IL-33 expression to a greater degree than IL-1*β* and tumor necrosis factor-*α*. dsRNA induced IL-33 expression via the TLR3-p38 mitogen-activated protein kinase-nuclear factor-*κ*B (NF-*κ*B) pathway. In addition, formation of the p65 and peroxisome proliferator-activated receptor*-γ* transcriptional complex was required for dsRNA-induced IL-33 expression. IL-33, in turn, acted on chondrocytes to induce matrix metalloproteinase-1/13 and inhibit type II collagen expression. These findings reveal that dsRNA released from damaged articular chondrocytes promotes cartilage degeneration via the TLR3-IL-33 pathway.

Osteoarthritis (OA) is a chronic, non-classical inflammatory disease of the diarthrodial synovial joints and a leading cause of disability in the adult population worldwide. Long-term, abnormal mechanical stress on the articular cartilage is a primary reason for the development of OA, but genetic and environmental factors also have critical roles in its pathogenesis.^1^ Progressive loss of collagens and proteoglycans is caused by the concerted activity of matrix metalloproteases (MMPs) and aggrecan-degrading enzymes.^2^ Specifically, MMP-1, MMP-3, MMP-13, a disintegrin and metalloproteinase (ADAM) with thrombospondin motifs 4 (ADAMTS4), and ADAM5 have been implicated in OA pathology.^3^ Proinflammatory cytokines, such as interleukin-1*β* (IL-1*β*), tumor necrosis factor-*α* (TNF-*α*), and IL-6, are believed to have important roles in the pathophysiology of OA by upregulating MMP expression in synovial cells and chondrocytes, and they may drive cartilage damage in OA.^2, 4, 5, 6^ However, blocking IL-1 and/or TNF-*α* does not lead to complete protection of the joint structure, indicating that other signaling pathways that mediate joint catabolism remain to be elucidated.^7^

IL-33, also called IL-1F11, is a new member of the IL-1 family of cytokines that induces signal transduction by binding to the heterodimeric receptor complex consisting of ST2 and IL-1 receptor accessory protein.^8^ IL-33 was reported to be expressed by endothelial and epithelial cells after stress, and it was released by the cells as an alarm to initiate inflammatory responses.^9^ Recently emerging evidence has shown that IL-33 is crucial in protection from parasitic infections and skin *Staphylococcus aureus* infection.^10, 11, 12, 13^ In addition to its role as a proinflammatory cytokine in the pathogenesis of asthma, atopic dermatitis, and allergic shock, all diseases characterized by Th2 inflammatory responses,^8, 9, 14, 15^ IL-33 was also found to be essential for the initiation of rheumatoid arthritis, which is an autoimmune bone disease characterized by Th1/Th17 responses.^16, 17^ However, the function of IL-33 in another bone disease, OA, is not well understood.

Recent studies have demonstrated that Toll-like receptors (TLRs), which are activated by damage-associated molecular patterns, might take part in the etiology of OA.^7, 18^ Ten members of the TLR family have been identified. They recognize a variety of molecules, ranging from viral- or endogenous-derived RNA (TLR3, -7, and -8) and DNA (TLR9) to bacterial-derived lipoproteins (TLR1, -2, and -6), lipopolysaccharide (TLR4), and flagellin (TLR5).^18^ In OA, the damage-associated molecular patterns, which consist of extracellular matrix molecules including the glycosaminoglycan hyaluronan, calcium pyrophosphate, and sodium urate crystals, can bind to chondrocyte TLRs and may, therefore, have a part in the etiology of OA.^19, 20^ However, the requirement of TLR activation for long-term, abnormal mechanical stress-induced OA responses needs further investigation.

In the present study, we set out to detect whether IL-33 is upregulated in osteoarthritic cartilage and determine whether it exhibits a functional relevance in OA, as well as to investigate if TLR3 activation is involved in long-term, abnormal mechanical stress-induced OA responses. Our findings uncover a vital role of the TLR3-IL-33 pathway in mechanical stress-induced OA and delineate a previously unknown mechanism in this disease.

## Results

### IL-33 expression is increased in the articular chondrocytes of osteoarthritic patients

**Figure 1:**
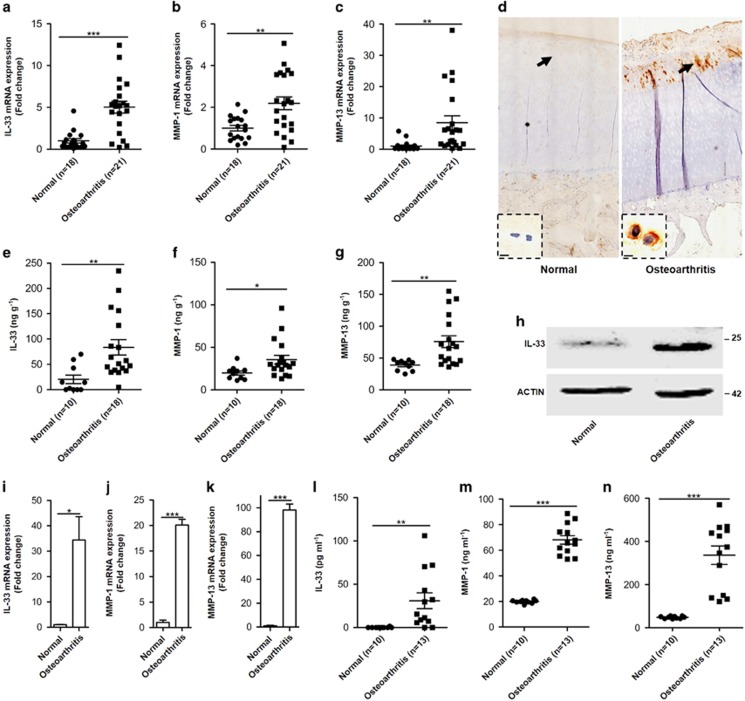
IL-33 expression is increased in the articular chondrocytes of osteoarthritic patients. (**a**–**c**) Quantification of *Il-33* (**a**), *Mmp-1* (**b**), and *Mmp-13* (**c**) expression in articular tissues from normal and osteoarthritic patients. (**d**) Immunohistochemical analysis of IL-33 expression in articular tissue from normal and osteoarthritic patients. Scale bar represents 20 *μ*m. (**e**–**g**) Quantification of IL-33 (**e**), MMP-1 (**f**), and MMP-13 (**g**) in normal and osteoarthritic cartilages detected by ELISA. (**h**) Western blot of IL-33 in normal and osteoarthritic cartilages. (**i**–**k**) Quantification of *Il-33* (**i**), *Mmp-1* (**j**), and *Mmp-13* (**k**) expression in normal and osteoarthritic cartilages. (**l**–**n**) Quantification of IL-33 (**l**), MMP-1 (**m**), and MMP-13 (**n**) in synovial fluid of normal and osteoarthritic patients detected by ELISA. *P<0.05, **P<0.01, and ***P<0.001. *P*-values were analyzed by *t*-test

To explore the role of IL-33 in OA, we first evaluated IL-33 expression in the normal articular tissues collected from 18 patients with fracture of tibial plateau and osteoarthritic articular tissues collected from 21 patients underwent total knee arthroplasty (TKA) surgery for end-stage OA of knees, respectively. The quantitative mRNA expression detected by real-time reverse transcription-polymerase chain reaction (RT-PCR) showed that *Il-33* (Figure 1a), *Mmp-1* (Figure 1b), and *Mmp-13* (Figure 1c) were significantly increased in the osteoarthritic articular cartilage compared with the normal controls. IL-33 protein expression was detectable by immunohistochemical staining that was primarily localized to the articular chondrocytes (Figure 1d). To further confirm that IL-33 was mainly expressed by chondrocytes, we collected cartilages from normal and osteoarthritic joints and measured the protein level by enzyme-linked immunosorbent assay (ELISA) (Figure 1e) or western blot (Figure 1h), and the mRNA expression by real-time RT-PCR (Figure 1i); these assays all confirmed that IL-33 was mainly induced in osteoarthritic chondrocytes over normal controls. Consistent with the IL-33 expression, MMP-1 (Figures 1f and j) and MMP-13 (Figures 1g and k) expression were significantly upregulated in the osteoarthritic cartilages compared with the normal controls. In addition to being highly expressed in chondrocytes, IL-33 (Figure 1l), MMP-1 (Figure 1m), and MMP-13 (Figure 1n) were all more easily detected in the synovial fluid of osteoarthritic patients than that from normal controls. Taken together, these data demonstrated that IL-33 expression was abundantly upregulated in osteoarthritic chondrocytes.

### Mechanical stretch induces IL-33 expression in articular chondrocytes

**Figure 2:**
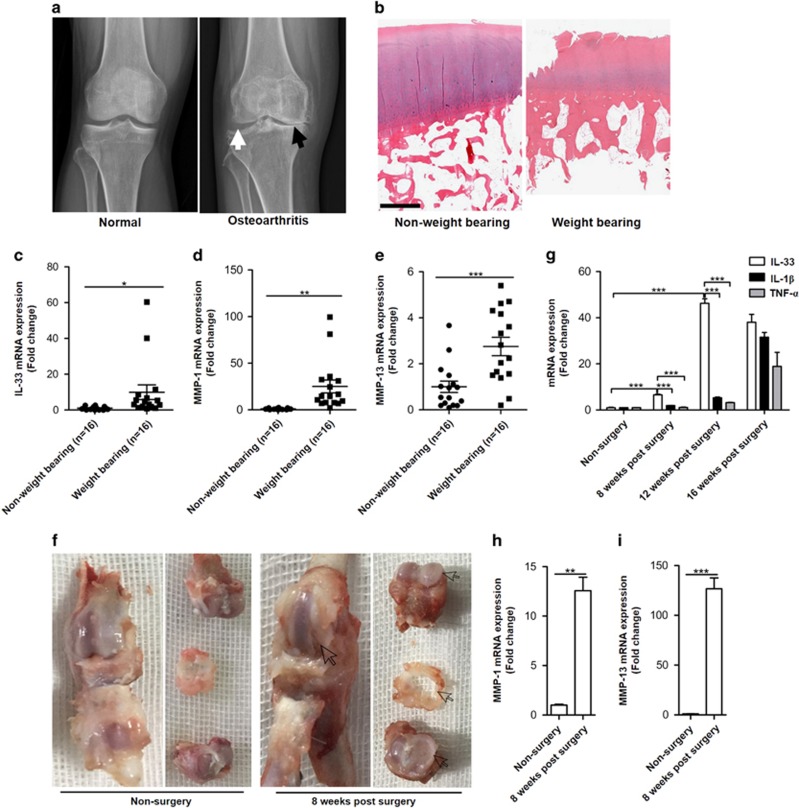
Mechanical stretch induces IL-33 expression in articular chondrocytes. (**a**) Plain radiographs of normal (left) and osteoarthritic (right) knee; white arrow indicates non-weight-bearing area, while black arrow indicates weight-bearing area. (**b**) Histological images of knee articular tissue stained with H&E. Scale bar represents 50 *μ*m. (**c**–**e**) Quantification of *Il-33* (**c**), *Mmp-1* (**d**), and *Mmp-13* (**e**) expression in the articular cartilages of weight-bearing and non-weight-bearing areas. (**f**) Representative macroscopic images of rat knees with or without DMM surgery; the arrows indicate cartilage erosion and medial meniscus wear. (**g**–**i**) Quantification of *Il-33*, *Il-1β*, and *Tnf-α* (**g**); *Mmp-1* (**h**); and *Mmp-13* (**i**) expression in normal and osteoarthritic rat articular chondrocytes. **P<0.01 and ***P<0.001. *P*-values were analyzed by two-way analysis of variance (ANOVA) in (**g**) and by *t*-test in (**c–e**, **h**, and **i**)

An increasing body of work has established that long-term, unbalanced mechanical stretch stress on articular cartilage is a primary risk factor for OA.^14, 20, 21, 22, 23, 24^ The knee X-ray radiographic images (Figure 2a) and hematoxylin and eosin (H&E) staining (Figure 2b) of osteoarthritic knee articular cartilage in our study also showed more progressive cartilage degradation and destruction in the weight-bearing cartilages (indicated by black arrow) than that in non-weight-bearing cartilages (indicated by white arrow). Therefore, to further investigate whether the increased IL-33 production in cartilage chondrocytes had a causal relationship with OA, we first detected IL-33 expression in the cartilage from weight-bearing and non-weight-bearing areas. The quantitative mRNA expression results showed that *Il-33* (Figure 2c), *Mmp-1* (Figure 2d), and *Mmp-13* (Figure 2e) were notably increased in the cartilage of weight-bearing areas compared with that in the cartilage of non-weight-bearing areas. Then, we used the destabilized medial meniscus (DMM)-induced experimental OA model in rats. We found that, 8 weeks after surgery, articular cartilage erosion was detectable in the medial condyle of the DMM knee (arrowheads indicated) and that the medial meniscus was distinctly worn, whereas the cartilage and medial meniscus of the non-surgery knee were intact (Figure 2f). Consistent with the morphological results, *Il-33* (Figure 2g), *Mmp-1* (Figure 2h), and *Mmp-13* (Figure 2i) levels were all markedly increased in articular cartilages after surgery; *Il-33* expression was upregulated within 8 weeks, peaked at 12 weeks, and remained elevated until week 16 (Figure 2g). As IL-1*β* and TNF-*α* are known to have crucial roles in cartilage extracellular matrix degradation in the pathogenesis of OA, we also assessed *Il-1β* and *Tnf-α* expression. We found that, unlike *Il-33*, *Il-1β*, and *Tnf-α* expression was induced at the late stage of the DMM-induced OA model, as expression levels were significantly increased only at 16 weeks after surgery (Figure 2g). Taken together, the results showed that long-term and unbalanced mechanical stretch stress induced IL-33 expression in articular chondrocytes.

### dsRNA released from damaged cartilages activates TLR3 to induce IL-33 expression

**Figure 3:**
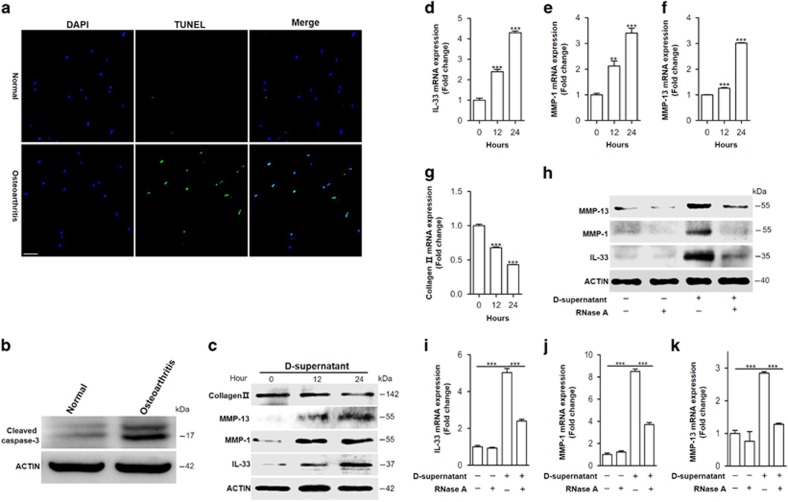
RNAs released from injured osteoarthritic cartilages induce IL-33 expression in normal human chondrocytes. (**a**) TUNEL immunofluorescent staining in normal and osteoarthritic cartilage tissue slices. Scale bar represents 50 *μ*m. (**b**) Western blot analysis of cleaved caspase-3 in normal and osteoarthritic cartilages. Samples from four OA patients were pooled together. (**c**) Western blot for IL-33, MMP-1, MMP-13, and type II collagen expression in normal human chondrocytes treated with the supernatants of osteoarthritic cartilage tissue homogenates. D-supernatant represents supernatant from damaged cartilage lysate. (**d**–**g**) Quantification of *Il-33* (**d**), *Mmp*-1 (**e**), and *Mmp*-13 (**f**) and *collagen II* (**g**) expression in normal human chondrocytes induced by the supernatant of damaged cartilage lysate at different time points. (**h**) Western blot analysis for IL-33, MMP-1, and MMP-13 induced by the supernatant of osteoarthritic cartilage tissue homogenates in human chondrocytes pretreated with or without 5 *μ*g/ml RNase A. D-supernatant represents supernatant from damaged cartilage lysate. (**i**–**k**) Quantification of *Il-33* (**i**), *Mmp*-1 (**j**), and *Mmp*-13 (**k**) expression in normal human chondrocytes induced by the supernatant of osteoarthritic cartilage tissue homogenates in human chondrocytes pretreated with or without 5 *μ*g/ml RNase A. D-supernatant represents supernatant from damaged cartilage lysate. **P<0.01 and ***P<0.001. *P*-values were analyzed by one-way ANOVA

**Figure 4:**
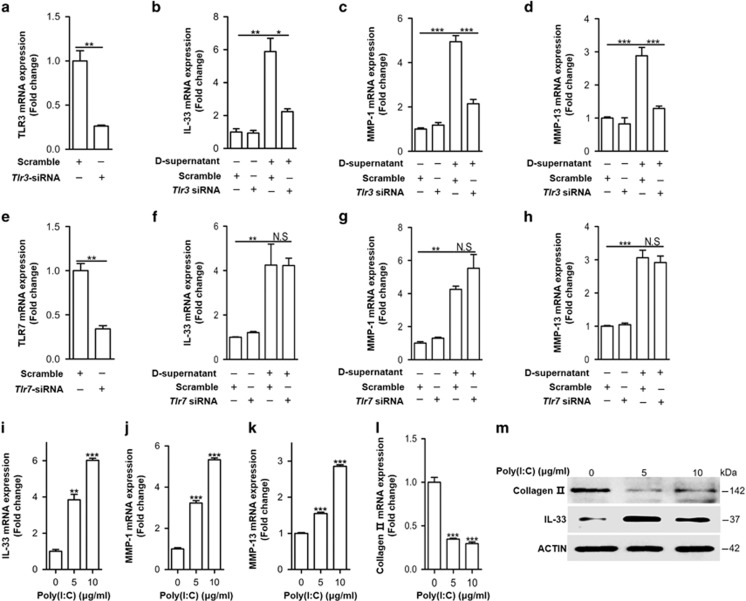
dsRNA released from damaged cartilages activates TLR3 to induce IL-33 expression. (**a**–**d**) Quantification of *Il-33* (**b**), *Mmp*-1 (**c**), and *Mmp*-13 (**d**) expression in normal human chondrocytes induced by the supernatant of osteoarthritic cartilage tissue homogenates in human chondrocytes transfected with or without *Tlr3*-siRNA. D-supernatant represents supernatant from damaged cartilage lysate. (**e**–**h**) Quantification of *Il-33* (**f**), *Mmp*-1 (**g**), and *Mmp*-13 (**h**) expression in normal human chondrocytes induced by the supernatant of osteoarthritic cartilage tissue homogenates in human chondrocytes transfected with or without *Tlr7* siRNA. D-supernatant represents supernatant from damaged cartilage lysate. (**i**–**l**) Quantification of *Il-33* (**i**), *Mmp*-1 (**j**), *Mmp*-13 (**k**) and *collagen II* (**l**) expression in normal human chondrocytes induced by different doses of poly(I:C) for 24 h. (**m**) Western blot analysis of IL-33 and type II collagen in human chondrocytes induced by different doses of poly(I:C) for 24 h. *P<0.05, **P<0.01, and ***P<0.001. *P*-values were analyzed by one-way ANOVA

We next sought to identify the underlying mechanism by which IL-33 was regulated in chondrocytes. Endogenous double-stranded RNA (dsRNA) released from injured or necrotic cells is a potent activator of pattern recognition receptors (PRRs) in various cell types, including synovial fibroblasts and articular chondrocyes.^1, 25, 26, 27^ Thus, we hypothesized that dsRNA released from the injured articular chondrocytes could induce IL-33 expression. To test our hypothesis, we first examined cell death in the injured cartilage tissues. The immunofluorescent terminal deoxynucleotidyl transferase-mediated dUTP nick-end labeling (TUNEL) staining analysis (Figure 3a) and western blot result of cleaved caspase-3 (Figure 3b) showed more cell death in the damaged osteoarthritic cartilages than in the normal cartilages. Then, we stimulated the normal primary human chondrocytes with supernatants from the damaged and healthy cartilage lysates. The supernatant of damaged but not healthy cartilage lysates significantly induced IL-33, MMP-1, and MMP-13 expression, as well as inhibited type II collagen expression (Figures 3c–g and Supplementary Figure S1). However, these processes were markedly dampened by 5 *μ*g/ml RNase A (Figures 3h–k) incubation. These findings indicated that there were RNAs released from the injured articular cartilages and increased IL-33 expression, as RNase A cleaves single- and double-stranded RNA, as well the RNA strand in RNA–DNA hybrids at low salt concentrations.^28^ TLR3 has been demonstrated to signal in response to dsRNA but not single-stranded RNA (ssRNA), while TLR7 has a critical role in viral ssRNA rather than dsRNA recognition.^29, 30^ Thereby, to determine whether dsRNA or ssRNA in the supernatant worked as a main stimulus for increased IL-33 expression, we knocked down *Tlr3* or *Tlr7* with siRNA, respectively, and detected IL-33 expression in the primary human chondrocytes stimulated with the supernatant of damaged cartilage lysates. The results revealed that *Tlr3*, rather than *Tlr7* knockdown, significantly dampened the increased IL-33, MMP-1, and MMP-13 expression (Figures 4a–h). Moreover, the expression of IL-33, MMP-1, MMP-13, and collagen II in response to commercial dsRNA analog poly(I:C) or commercial ssRNA derived from HIV-1 further demonstrated that it was dsRNA rather than ssRNA released from damaged cartilages worked as a main inducer for IL-33 expression in human chondrocytes (Figures 4i–m and Supplementary Figure S2).

### p38 MAPK-NF-*κ*B are the downstream pathways of TLR3 activated by dsRNA

**Figure 5:**
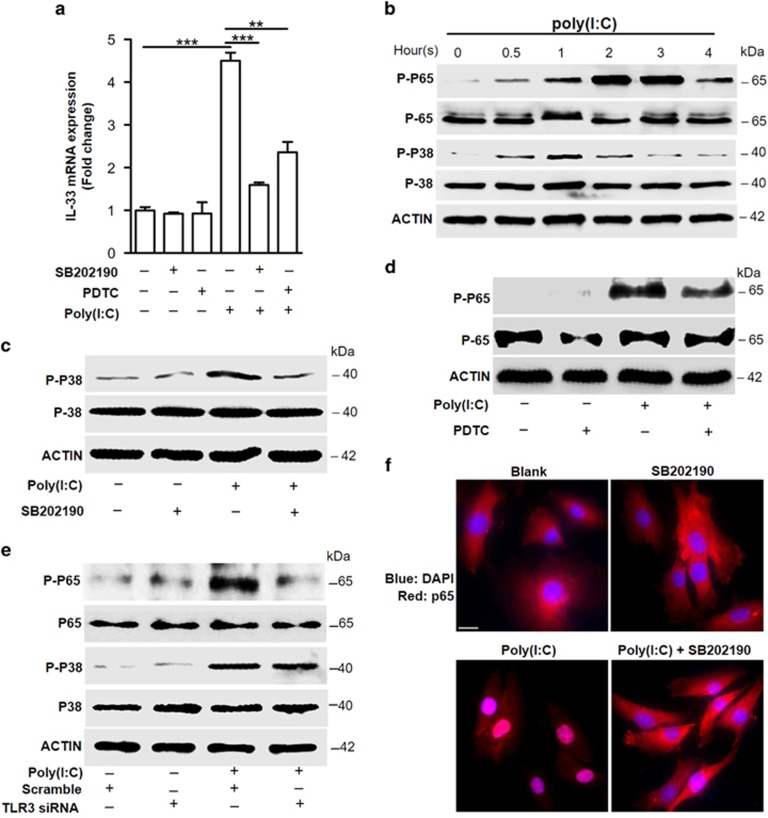
p38 MAPK-NF-*κ*B are the downstream pathways of TLR3 activated by dsRNA. (**a**) Quantification of IL-33 mRNA expression in human chondrocytes induced by 10 *μ*g/ml poly(I:C) in the presence of the p38 MAPK inhibitor SB202190 or the p65 inhibitor PDTC. (**b**) Phosphorylation of p38 MAPK (Thr180/Tyr182) and p65 (Ser536) in human chondrocytes after treatment with 10 *μ*g/ml poly(I:C). (**c** and **d**) Phosphorylation of p38 MAPK (**c**) and p65 (**d**) induced by 10 *μ*g/ml poly(I:C) in the presence of the p38 MAPK inhibitor SB202190 or the p65 inhibitor PDTC. (**e**) Phosphorylation of p38 MAPK and p65 in human chondrocytes induced by 10 *μ*g/ml poly(I:C) with or without *Tlr3* knockdown. (**f**) Immunofluorescent staining of p65 in normal human chondrocytes treated with 10 *μ*g/ml poly(I:C) in the presence or absence of the p38 MAPK inhibitor SB202190. Scale bar represents 50 *μ*m. ***P<0.001. *P*-values were analyzed by one-way ANOVA

Having observed dsRNA released from the damaged cartilages works as a main stimulus for IL-33 and which increased IL-33 expression through TLR3 pathway, we next sought to explore the dowmstream pathways of TLR3 activated by dsRNA in chondrocytes. Inhibitors of several key pathways were used to pretreat chondrocytes in the presence of poly(I:C). Among these inhibitors, p38 mitogen-activated protein kinase (p38 MAPK) inhibitor SB202190 and the p65 inhibitor PDTC significantly decreased the *Il-33* expression induced by 10 *μ*g/ml poly(I:C) (Figure 5a). Poly(I:C) induced phosphorylation of p38 MAPK and p65 in a time-dependent manner (Figure 5b), and the phosphorylation of p38 MAPK and p65 induced by poly(I:C) were dampened by the p38 MAPK and p65 inhibitors, respectively (Figures 5c and d). Furthermore, poly(I:C)-induced p38 MAPK and p65 phosphorylation were significantly decreased after *Tlr3* was silenced in chondrocytes (Figure 5e). These data demonstrated that p38 MAPK and p65 were the key downstream molecules of TLR3 that participated in dsRNA-induced IL-33 expression. Finally, immunofluorescent staining demonstrated that p65 is downstream of p38 in the poly(I:C)-activated TLR3 pathway, as the p38 MAPK inhibitor SB202190 significantly inhibited poly(I:C)-induced p65 translocation (Figure 5f). Taken together, these data demonstrate that dsRNA induces IL-33 expression via TLR3-p38 MAPK-NF-*κ*B pathway.

### The formation of p65 and PPAR*γ* transcriptional complex is required for dsRNA-induced IL-33 expression

**Figure 6:**
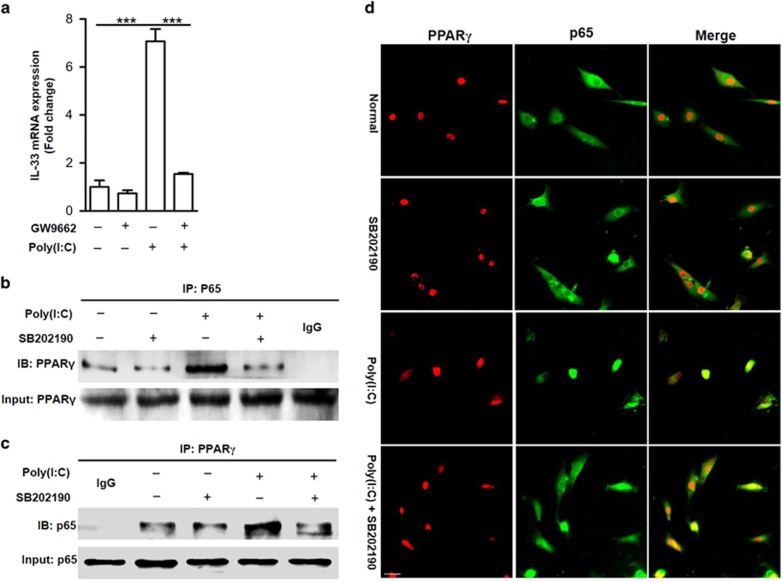
p65 and PPAR*γ* transcriptional complex formation is required for dsRNA-induced IL-33 expression. (**a**) Quantification of IL-33 mRNA expression induced by 10 *μ*g/ml poly(I:C) in the presence of the PPAR*γ* inhibitor GW9662. (**b**,**c**) Immunoprecipitation of p65 and PPAR*γ* in human chondrocytes induced by 10 *μ*g/ml poly(I:C) pretreated with or without the p38 MAPK inhibitor SB202190. (**d**) Immunofluorescent staining of PPAR*γ* and p65 in normal human chondrocytes induced by 10 *μ*g/ml poly(I:C) in the presence of the p38 MAPK inhibitor SB202190. Scale bar represents 50 *μ*m. ***P<0.001. *P*-values were analyzed by one-way ANOVA

The peroxisome proliferator-activated receptor-*γ* (PPAR*γ*) is a member of the nuclear receptor superfamily that activates target gene transcription in a ligand-dependent or -independent manner.^31, 32^ In addition, PPAR*γ* has been suggested to be involved in a broad range of cellular functions by interacting with p65.^33, 34^ Therefore, we hypothesized that PPAR*γ* might participate in poly(I:C)-induced IL-33 expression by interacting with p65. To test this, we first detected IL-33 expression induced by poly(I:C) in the presence or absence of the PPAR*γ* inhibitor GW9662. The results showed that GW9662 significantly inhibited poly(I:C)-induced *Il-33* expression in normal human chondrocytes (Figure 6a). Furthermore, we found that poly(I:C) induced the formation of the p65 and PPAR*γ* transcriptional complex in chondrocytes, but this interaction was dampened by the p38 MAPK inhibitor SB202190 (Figures 6b–d); these results further confirmed that p65 was downstream of p38 MAPK in the poly(I:C)-induced IL-33 pathway.

### IL-33 mediates osteoarthritic cartilage degradation by regulating MMP-1 and MMP-13 expression

**Figure 7:**
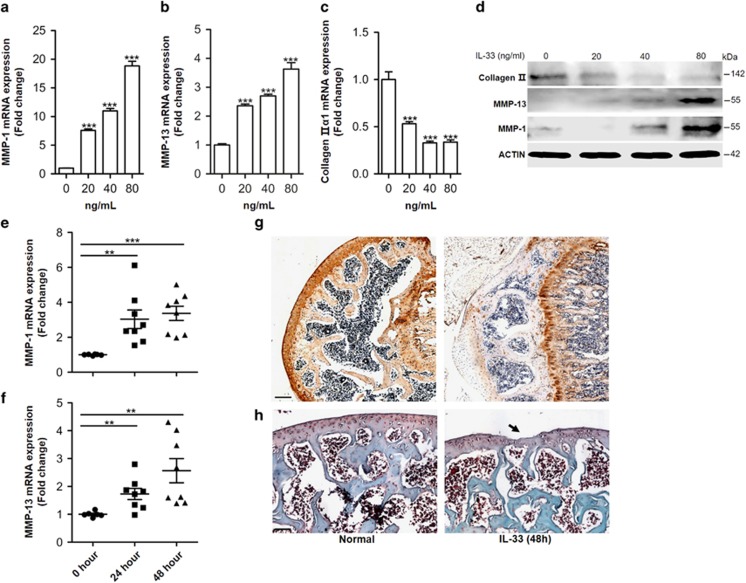
IL-33 increases MMP-1 and MMP-13 expression in chondrocytes. (**a**–**c**) Dose-dependent expression of *Mmp-1* (**a**), *Mmp-13* (**b**), and *type II collagen* (**c**) induced by IL-33 in human chondrocytes. (**d**) Western blot of MMP-1, MMP-13, and type II collagen expression in human chondrocytes induced by different doses of IL-33. (**e** and **f**) Quantification of *Mmp-1* (**e**) and *Mmp-13* (**f**) expression in mouse cartilage tissues 24 or 48 h after IL-33 intra-articular injection. (**g**) Immunohistochemical analysis of type II collagen expression in mouse articular tissues 48 h after IL-33 intra-articular injection. (**h**) Safranin O staining of mouse articular tissues 48 h after IL-33 intra-articular injection. Scale bar represents 100 *μ*m. **P<0.01 and ***P<0.001. *P*-values were analyzed by one-way ANOVA

We next set out to elucidate the biological role of IL-33 in OA. Previous observations have suggested that IL-1*β* induces MMP expression to promote cartilage extracellular matrix degradation,^2, 20, 35^ and that MMP-1 and MMP-13 appear to be the most important of the 20 members of the MMP family in cartilage degradation during OA;^35, 36, 37^ thus, we hypothesized that IL-33 might be involved in the pathogenesis of OA by regulating MMP-1 and MMP-13 expression. To test our hypothesis, we first stimulated chondrocytes with recombinant IL-33 *in vitro* and found that MMP-1 and MMP-13 were induced by IL-33 at both the mRNA and protein level in a dose-dependent manner (Figures 7a, b, and d); however, type II collagen expression was significantly inhibited (Figures 7c and d). Furthermore, in addition to increasing *Mmp-1* and *Mmp-13* expression (Figures 7e and f), IL-33 intra-articular injection significantly inhibited type II collagen expression (Figure 7g) and induced aggressive cartilage destruction (Figure 7h) *in vivo*. Taken together, these data demonstrated that IL-33 could mediate osteoarthritic cartilage degradation directly by regulating MMP-1 and MMP-13 expression.

**Figure 8:**
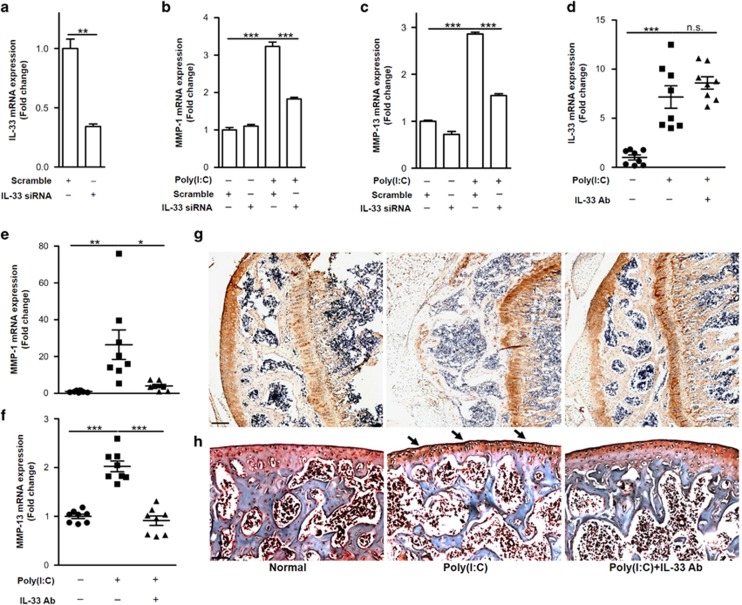
IL-33 is the mediator of dsRNA-induced MMP-1 and MMP-13 expression. (**a**) IL-33 expression in normal human chondrocytes treated with or without *Il-33* siRNA. (**b** and **c**) *Mmp-1* and *Mmp-13* expression induced by 10 *μ*g/ml poly(I:C) before or after *Il-33* was silenced in human chondrocytes. (**d**–**f**) Quantification of *Il-33* (**d**), *Mmp-1* (**e**), and *Mmp-13* (**f**) expression in mouse cartilage induced by poly(I:C) intra-articular injection in the presence or absence of an IL-33-neutralizing antibody. (**g**) Immunohistochemical analysis of type II collagen expression in mouse articular tissues in the presence or absence of an IL-33-neutralizing antibody. Scale bar represents 100 *μ*m. (**h**) Safranin O staining of mouse articular tissues induced by poly(I:C) intra-articular injection in the presence or absence of an IL-33-neutralizing antibody. Scale bar represents 100 *μ*m. *P<0.05, **P<0.01 and ***P<0.001. *P*-values were analyzed by *t*-test in (a), one-way ANOVA in (**b**–**f**)

Since our previous data demonstrated that dsRNA induced MMP-1 and MMP-13, as well as IL-33 expression, we then investigated if IL-33 worked as a mediator of dsRNA-induced MMP-1 and MMP-13 expression. The results showed that poly(I:C) significantly induced *Mmp-1* and *Mmp-13* expression, and this induction was significantly inhibited after *Il-33* was silenced with *Il-33* siRNA (Figures 8a–c) in human primary chondrocytes. As previous works have reported that intra-articular injection of dsRNA led to the induction of arthritis,^38, 39^ we next sought to investigate the role of IL-33 in the pathogenesis of a dsRNA-induced arthritic responses. We initiated the arthritis model by intra-articular injection of poly(I:C). The results showed that *Il-33*, *Mmp-1*, and *Mmp-13* were abundantly increased in articular cartilage 48 h after poly(I:C) intra-articular injection (Figures 8d–f). However, these effects were significantly dampened by intra-articular injection of an IL-33-neutralizing antibody. In addition, poly(I:C)-inhibited type II collagen expression was restored after IL-33 activity was blocked (Figure 8g). Consistent with the effects on MMP-1 and MMP-13 expression, poly(I:C)-induced cartilage degradation and destruction were significantly improved in the group that received intra-articular injection of the IL-33-neutralizing antibody compared with the controls (Figure 8h). Collectively, these data demonstrated that IL-33-mediated osteoarthritic cartilage degradation by regulating MMP-1 and MMP-13 expression.

## Discussion

OA results in the destruction of cartilage and subchondral bone, ultimately leading to the loss of joint function. Chondrocytes respond to a variety of stimuli, such as proinflammatory cytokines and mechanical loading, by expressing degradative enzymes and catabolic mediators. Whether the initiation of matrix degradation is cytokine driven or biomechanical, it is likely that the downstream degradative pathways are similar, involving the upregulating of specific MMPs, especially MMP-1 and MMP-13, which have been demonstrated to be the most important in cartilage extracellular matrix degradation.^35, 36, 37, 40^ Here, we observed that IL-33 promoted cartilage degeneration by inducing MMP-1 and MMP-13 expression in chondrocytes. Our results demonstrated that dsRNA released from injured chondrocytes increased IL-33 expression via activation of TLR3-p38 MAPK-NF-*κ*B pathway. Furthermore, p65 and PPAR*γ* transcriptional complex formation is required for dsRNA-induced IL-33 expression. Therefore, the identification of IL-33 as a stimulus of MMP-1 and MMP-13, and the elucidation of its mechanisms of induction and action, provide new insights into the promotion of arthritis responses by the innate immune system. These findings also offer potential targets for the treatment of arthritis responses.

Defining the pathogenesis of cartilage degradation in OA is a continuing challenge, but several studies have demonstrated that inflammatory cytokines, such as IL-1*β*, IL-6, and TNF-*α*, are not only upregulated in OA joint cartilage but also found at high levels in the synovial fluid of OA patients compared with that of unaffected patients. They are believed to have important roles in the pathophysiology of OA by upregulating the expression of matrix-degrading MMPs and aggrecanases, and downregulating the synthesis of type II collagen and aggrecan, which are the building blocks of cartilage matrix.^2, 6, 41, 42, 43^ However, studies have also suggested that blocking IL-1 and/or TNF-*α* does not lead to complete protection of the joint structure, indicating that other signaling pathways that mediate joint catabolism remain to be elucidated.^7^ Here, we observed that the mRNA and protein expression of IL-33 are increased in the articular cartilage of OA patients compared with unaffected controls. We also found that IL-33 is preferentially expressed in the chondrocytes of weight-bearing areas compared with non-weight-bearing areas, which was confirmed in DMM-induced experimental OA, a model that results from long-term, unbalanced stress loaded on the articular cartilage.^44, 45, 46^ Interestingly, we found that, although IL-33, IL-1*β*, and TNF-*α* are all upregulated in the cartilage during DMM-induced OA, IL-33 is upregulated within 8 weeks, peaks at 12 weeks, and remains elevated until 16 weeks after surgery. However, IL-1*β* and TNF-*α* are significantly increased only 16 weeks after surgery. Therefore, compared with IL-1*β* and TNF-*α*, IL-33 may work as an alarm at the initial stage of the host immune response to long-term, unbalanced stress loaded on the chondrocytes. In addition, IL-33, MMP-1, and MMP-13 are found at high levels in the synovial fluid of OA patients, but are nearly undetectable in normal synovial fluid. Thus, in the pathogenesis of OA, IL-33 expression is upregulated and induces chondrocytes to express MMP-1 and MMP-13, thereby promoting cartilage degradation and destruction.

The catabolic triggers of articular chondrocytes for cartilage degradation in the pathogenesis of OA have not been established; however, it has been reported that cartilage cell death due to aging or exposure to mechanical stress and the activation of abnormal gene expression patterns initiate the cartilage remodeling process that ultimately leads to OA pathology and symptoms.^1, 25, 47^ Mounting evidence in recent studies suggests that endogenous dsRNA released from injured or necrotic cells is a potent activator of PRRs in various cell types, including synovial fibroblasts and articular chondrocyes.^1, 25, 26, 27^ Our findings confirmed that long-term, unbalanced stress induces cartilage damage,^2, 48^ and suggested that dsRNA derived from damaged cartilage activate TLR3 to induce IL-33 expression. IL-33, in turn, acts on chondrocytes to promote cartilage degradation by increasing MMP-1 and MMP-13 expression; the role of dsRNA was supported by the fact that RNase A pretreatment of the supernatants from damaged osteoarthritic cartilage homogenates significantly dampened IL-33, MMP-1, and MMP-13 expression. In addition, dsRNA intra-articular injection induced abundant IL-33, MMP-1, and MMP-13 expression along with OA symptoms. This is consistent with reports that the activation of TLR3 by dsRNA released from necrotic cells potently induces MMP-13 in chondrocytes,^49, 50^ and that the injection of dsRNA into mice resulted in a self-limited arthritis.^38, 50^ Finally, our data demonstrate that IL-33 is the mediator of dsRNA-induced MMP-1 and MMP-13 expression; dsRNA-induced MMP-1 and MMP-13 expression was significantly dampened after *Il-33* silencing in chondrocytes; in addition, dsRNA intra-articular injection induced MMP-1 and MMP-13 expression, and articular degradation was significantly decreased after IL-33 activity was blocked with an IL-33-neutralizing antibody.

PPAR*γ* is a member of the nuclear hormone receptor superfamily that is capable of both positive and negative regulation of gene expression in ligand-dependent and -independent manners.^31, 32^ PPAR*γ* has been suggested to be involved in a series of cellular functions, including adipocyte differentiation, inflammatory responses, and apoptosis;^31^ however, there are few reports on the function of PPAR*γ* in the pathogenesis of arthritis. Here, our results show that, in addition to p38 MAPK phosphorylation and NF-*κ*B (p65) translocation, PPAR*γ* activation and formation of a transcriptional complex with p65 are also required for dsRNA-induced IL-33 expression. In addition, a p38 MAPK inhibitor blocked p65 translocation, which further demonstrates that NF-*κ*B is downstream of p38 MAPK in the dsRNA-activated TLR3 pathway. Although PPAR*γ* has been reported to participate in anti-inflammatory responses, and to block bone resorption by interacting with p65 under interaction with its ligands.^33, 34^ Some groups have also found that PPAR*γ* acts as a double-edged sword, in that it promotes inflammatory responses in a ligand-independent manner, whereas it serves as a negative regulator of renal inflammation upon ligand activation.^32^ Our results provide new evidence that PPAR*γ* is a positive mediator in inflammatory responses in a ligand-independent manner.

However, we would like to point out some potential limitations of our study. First, the concentration of total RNAs in the supertanants of damaged cartilage lysate is ~24.8 ng/*μ*l quantified by using a Qubit^3^ Fluorometer (Invitrogen, Grand Island, NY, USA); however, because of technology limitation, we cannot measure the exact concent of dsRNA within these total RNAs. Second, although our results revealed that dsRNA induced IL-33 expression via the TLR3-p38 MAPK-NF-*κ*B p65 pathway, as well as required the formation of the p65 and PPAR*γ* transcriptional complex, these results were acquired in *in vitro* experiments; thus, the exact induction mechanisms of IL-33 *in vivo* still require further investigation. Second, although we observed increased expression of IL-33 in OA chondrocytes, as well as in the cartilage tissue in a DMM-induced OA model, the clinical relevance of IL-33 in OA still needs further investigation. Third, in addition to TLR3, the RIG-I-like receptors RIG-I and MDA-5 can also recognize dsRNA and its synthetic analog, poly(I-C); thus, it is unknown if RIG-I and MDA-5 are also involved in dsRNA-mediated IL-33 expression. Last, although reports suggest that MMP-1 and MMP-13 are the most important of the 20 members of the MMP family in cartilage degradation during OA,^35, 36, 37^ MMP-3, ADAMTS4, and ADAM5 have also been implicated in this process; therefore, the possible regulation of MMP-3, ADAMTS4, and ADAMTS5 expression by IL-33 during OA is a path for further investigation.

In summary, our study provides evidence that IL-33 mediates arthritic responses by regulating MMP-1 and MMP-13 expression. IL-33 is an intermediate molecule in the crosstalk between mechanical stress and innate immune responses in articular cartilage, and may function as a previously unknown key target for the treatment of arthritis.

## Materials and methods

### Normal and OA cartilage sample collection

Normal human cartilage samples obtained from 18 patients with fracture of tibial plateau. Osteoarthritic cartilage samples were obtained from 21 patients who underwent TKA surgery for end-stage OA of knees. At the same time, 0.2 ml synovial fluid was also collected from the articular cavity.

### Weight-bearing and non-weight-bearing area cartilage collection

Weight-bearing and non-weight-bearing area chondrocytes preparation was performed as reported.^2^ First, knee cartilages of 22 OA patients who underwent total joint arthroplasty were collected. Second, pieces of smooth cartilage were resected from the area with surface integrity without any irregularity (no staining with India ink, smooth cartilage) (non-weight-bearing area), while damaged cartilage was obtained from area with gross erosions and stained positive with India ink (weight-bearing area). Pieces of cartilage were stored in liquid nitrogen immediately for RNA isolation later.

### Mice and rats

Eight-week-old male C57BL/6 mice and Sprague–Dawley rats were purchased from Shanghai Laboratorial Animal Center, Chinese Academy of Sciences. The animals that were randomly grouped were not performed in a blinded manner. The animals were housed with free access to water and rat diet in an air-conditioned room with a 12-h light–dark cycle, at 21 °C to 23 °C and 60% relative humidity in the animal facility at Ruijin Hospital, Shanghai Jiaotong University (SJTU) School of Medicine, China.

### Ethics statement

All human sample acquisitions were approved by the ethical committee of Ruijin Hospital, SJTU School of Medicine, China, and performed in accordance with the Declaration of Helsinki Principles. All participants provided written informed consent, which was obtained before enrollment in the study. All animal experiments were performed according to the protocol approved by the SJTU Animal Care and Use Committee and in direct accordance with the Ministry of Science and Technology of the People’s Republic of China on Animal Care guidelines. All surgeries were performed under anesthesia and all efforts were made to minimize suffering.

### Primary chondrocyte monolayer culture and stimulation

Human full-thickness cartilage slices were obtained from above the subchondral bone of the cartilage from femoral neck fracture patients. Rat full-thickness cartilage slices were obtained from above the subchondral bone of the neonatal knee cartilage. For monolayer cultures, slices were minced and incubated sequentially with pronase and collagenase in Dulbecco’s modified Eagle’s medium (DMEM) until the fragments were digested. Released cells were seeded at 10^7^/plate in 10-cm culture plates in DMEM/F10 (Gibco, Sydney, NSW, Australia) medium containing 10% FBS (Gibco), 50 U/ml penicillin and 50 *μ*g/ml streptomycin (Gibco) under standard culture conditions. After 7 days, confluent chondrocytes were split once and seeded at high density, and these first-passage chondrocytes were authenticated by Alcian blue staining and mycoplasma contamination were tested before subsequent experiments. For all cell stimulation experiments, 10^5^ cells were seeded in each well of 24-well or 10^6^ cells were seeded in each well of 6-well plates. When cells were grown to 80% confluence, the indicated doses of recombinant human IL-33 (R&D, Abingdon, UK) or poly(I:C) (InvivoGen, San Diego, CA, USA) or different inhibitors under concentrations without cytotoxicity were used to stimulate cells. After 24 h, cells were collected for RNA isolation or western blot.

### Supernatant of cartilage homogenate preparation

One thousand milligram of damaged and healthy knee cartilage slices were abtained from five OA patients who underwent total joint arthroplasty and five patients with fracture of tibial plateau, separately. Slices were minced in 50 ml PBS and centrifuged at 12 000 r.p.m. for 15 min at 4 °C, and then the supertanant was collected and stored at −80 °C. RNA concentration in the supertanant of damaged cartilages lyste is 24.8 ng/*μ*l detected by Qubit 3 Fluorometer (Invitrogen), whereas the concentration in the healthy cartilage lysate was too low to detect. Before cell stimulation, the supernatant was pretreated with 5 *μ*g/ml RNase A for 30 min.

### DMM-induced experimental OA model

Experimental OA was induced using 8-week-old male Sprague–Dawley rats by DMM surgery (*n*=6; mean body weight, 240 g).^51^ After the rats were anesthetized, the right knee joint was exposed following a medial capsular incision and gentle lateral displacement of the knee extensor muscles without transection of the patellar ligament. Then, the medial meniscotibial ligament was transected, and the medial meniscus could be displaced medially. After replacement of the extensor muscles, the medial capsular incision was sutured, and the skin was closed. A sham operation was performed on the left knee joint using the same approach without medial meniscotibial ligament transection. The animals were then permitted unrestricted activity and provided free access to food and water. Knee joints were processed for histological and biochemical analysis 8 weeks after surgery.

### Double-stranded RNA intra-articular injection

Twenty microliters of (10 *μ*g/knee) dsRNA analog poly(I:C) was intra-articular injection according to the protocol reported before.^38^ The contralateral knee joints were injected with 20 *μ*l PBS and were always used as a negative control. Mice were killed 3 days later, articular cartilages were collected for RNA or protein collection or stored in 4% paraformaldehyde for histopathologic examination.

### IL-33 neutralization

Four micrograms of monoclonal mouse IL-33 antibody (R&D) was intra-peritoneally injected into each mouse 24 h before poly(I:C) infection. Next day, 4 *μ*g of monoclonal mouse IL-33 antibody (R&D) was intra-articularly injected. After 4 h, 20 *μ*l poly(I:C) was intra-articularly injected. Three days later, mice were killed and articular cartilages were collected.

### IL-33 intra-articular injection

Two micrograms of recombinant mouse IL-33 (R&D) in 20 *μ*l PBS was intra-articular injection, the contralateral knee joints were injected with 20 *μ*l PBS and were always used as a negative control. Mice were killed 24 or 48 h later, articular cartilages were collected for RNA or protein collection or stored in 4% paraformaldehyde for histopathologic examination.

### Real-time quantitative RT-PCR

Total RNA was prepared using Trizol reagent (Invitrogen) following the manufacturer’s instructions. Low yield of RNA due to technical problems were predetermined. RNA was quantified by Thermo NANODROP 2000 spectrophotometer (Pierce, Rockford, IL, USA). Total RNA (1 *μ*g) was reverse transcribed using GoScript Reverse Transcription System (cat. no.: A5001; Promega, Madison, WI, USA) according to the manufacturer’s instructions. QPCR were performed on Mx3005P (Stratagene, Cedar Creek, TX, USA) using GoTaq qPCR Master Mix (Promega; cat. no.: A6001/2). The following gene expression assays were purchased from Invitrogen: human glyceraldehyde 3-phosphate dehydrogenase (GAPDH) (NM_002046.5): 5′-CTTAGCACCCCTGGCCAAG-3′ (forward) and 5′-TGGTCATGAGTCCTTCCACG-3′ (reverse); human IL-33 (NM_033439.3): 5′-CAAAGAAGTTTGCCCCATGT-3′ (forward) and 5′-AAGGCAAAGCACTCCACAGT-3′ (reverse); human MMP-1 (NM_002421.3): 5′-ATTCTACTGATATCGGGGCTTTGA-3′ (forward) and 5′-ATGTCCTTGGGGTATCCGTGTAG-3′ (reverse); human MMP-13 (NM_002427.3): 5′-AGTTCGGCCACTCCTTAGGT (forward) and 5′-TGGTAATGGCATCAAGGGAT-3′ (reverse); human type II collagen (NM_001844.4): 5′-GCTCCCAGAACATCACCTACC-3′ (forward) and 5′-TGAACCTGCTATTGCCCTCT-3′ (reverse); human TLR3 (NM_003265.2): 5′-TGATGCTCCGAAGGGTGG-3′ (forward) and 5′-CAGGGTTTGCGTGTTTCC-3′ (reverse); human TLR7 (NM_016562.3): 5′-TCACCATTAACCACATACCA-3′ (forward) and 5′-GCCAGTTCTGTTAGATTCTC-3′ (reverse); mouse GAPDH (GU214026.1): 5′-CTTAGCCCCCCTGGCCAAG-3′ (forward) and 5′-TGGTCATGAGCCCTTCCACA-3′ (reverse); mouse IL-33 (NM_001164724.1): 5′-ATGGGAAGAAGCTGATGGTG-3′ (forward) and 5′-CCGAGGACTTTTTGTGAAGG-3′ (reverse); mouse MMP-1 (NM_032006.3): 5′-AGGTTTGGGGGGTGATG-3′ (forward) and 5′-TGGCTGGATGGGATTTG-3′ (reverse); mouse MMP-13 (NM_008607.2): 5′-CACAGCAAGCCAGAATAAAG-3′ (forward) and 5′-CACACATCAGTAAGCACCAAG-3′ (reverse); rat GAPDH (NM_017008.4): 5′-TCTACCCACGGCAAGTCC-3′ (forward) and 5′-GATGTTAGCGGGATCTCG-3′ (reverse); rat IL-33 (NM_001014166.1): 5′-GTGCAGGAAAGGAAGACTCG-3′ (forward) and 5′-TGGCCTCACCATAAGAAAGG-3′ (reverse); rat MMP-1 (NM_001134530.1): 5′-CACTCCCTTGGACTCACTCA-3′ (forward) and 5′-CCCATATAAAGCCTGGATGC-3′ (reverse); rat MMP-13 (NM_133530.1): 5′-TGGCGACAAAGTAGATGCTG-3′ (forward) and 5′-TGGCATGACTCTCACAATGC-3′ (reverse); rat type II collagen (NM_012929.1): 5′-CTCAAGTCGCTGAACAACCA-3′ (forward) and 5′-GTCTCCGCTCTTCCACTCTG-3′ (reverse). Quantification of gene expression was determined by the comparative 2^ΔΔCT^ method. The relative expression levels were determined by normalizing expression to glyceraldehyde 3-phosphate dehydrogenase (GAPDH). All the assays were performed in triplicate and repeated at least two times.

### Protein detection by ELISA

Articular cartilages were homogenized in 1 ml PBS using Ultra Turrax (IKA, Italy), the supernatant was harvested and assayed for cytokine conten using commercially available ELISA reagents for IL-33, MMP-1 and MMP-13 (Duoset R&D Systems, Abingdon, UK).

### Immunoprecipitation and immunoblotting

Chondrocytes treated with poly(I:C) (cat. no.: tlrl-picw; InvivoGen), IL-33 (cat. no.: 3625-IL; R&D) or SB202190 (cat. no.:S7067; Sigma, St Louis, MO, USA) was lysed with RIPA buffer (pH 7.4) containing protease inhibitor cocktail (Roche, Pleasanton, CA, USA). Low yield of protein due to technical problems were predetermined. Twenty micrograms of total protein was used for immunoprecipitation and 5 *μ*g of total protein was used for immunoblot. IL-33, p65, p38 MAPK, AKT, PPAR*γ*, MMP-1 and MMP-13 were detected by immunoblot with IL-33 antibody (cat. no.: AF3625; R&D), p65 (cat. no.: 6956; cat. no.: 8242; CST, Cambridge, MA, USA) and P-p65 (cat. no.: 3033; CST) antibody, p38 MAPK (cat. no.: 8690; CST) and P-p38 MAPK (cat. no.: 4511; CST) antibody, AKT (cat. no.: 4691; CST) and P-AKT (cat. no.: 4060; CST) antibody, PPAR*γ* antibody (cat. no.: 2443; CST), MMP-1 (cat. no.: ab46667; Abcam, Cambridge, MA, USA) and MMP-13 (cat. no.: ab77947; Abcam) antibody.

### Histological examination

The knee joints of rats and the samples from human articulars were fixed in 10% neutral-buffered formalin for 2 days, decalcified with 10% ethylene diamine tetra acetic acid (Sigma-Aldrich) at pH 7.4 for ~4 weeks, and then dehydrated in a graded ethanol solution series, embedded in paraffin, sliced consecutively into 5 *μ*m sections. Then, the sections were deparaffinized with xylene and hydrated, stained with hematoxylin and eosin (H&E), alcian blue and safranin O/fast green to examine the morphology and glycosaminoglycan content. Observation was performed under a light microscope (Zeiss, Jena, Germany).

### Immunohistochemistry staining

For immunohistochemistry, decalcificated articular cartilage sections were boiled in 10 mM sodium citrate (pH 6.0) for 5 min to retrieve antigen. Sections were quenched with 3% hydrogen peroxide for 15 min to reduce endogenous peroxidase activity and blocked with 3% normal goat serum in Tris-buffered saline. The sections were then incubated with goat anti-mouse IL-33 antibodies (R&D) or goat IgG as control at 4 °C overnight, followed by biotinylated secondary antibodies and a peroxidase-labeled streptavidin–biotin staining technique (DAB Kit; Invitrogen). Nuclei were counterstained with hemalum (FARCO Chemical Supplies, Hong Kong, China). The slides were visualized by a microscope (Zeiss, Axio, Germany).

### Immunofluoresent staining

Five micrometer of formalin-fixed, paraffin-embedded tissue sections or chondrocytes, which were cultured on polystyrene vessel tissue culture-treated glass slides (Becton Dickinson) were used for immunofluoresent staining. Two percent PFA was used to fixate the samples. After 10 min fixation and subsequent pretreated with antigen retrieval solution, the sections were stained with p65, PPAR*γ* and TUNEL antibody. The sections were reprobed with Alexa Fluor 488-conjugated goat anti-mouse (Invitrogen) or Alexa Fluor 594-conjugated goat anti-rabbit (Invitrogen), and then mounted in ProLong Gold antifade reagent with DAPI (Invitrogen) and visualized by the microscope (Leica).

### siRNA preparation and targeting gene knockdown

Oligonucleotides encoding human *Il-33*, *Tlr3* and *Tlr7* siRNA were designed and synthesized by GenePharma (Shanghai, China). Blast search was performed by using the National Center for Biotechnology Information (NCBI) database to ensure that siRNA constructs were targeting only human *Il-33*, *Tlr3* and *Tlr7*. *Il-33*, *Tlr3* and *Tlr7* siRNA construct and transfection were according to the manufacturer’s protocol of Lipofectamine 3000 (Invitrogen). After 24 h transfect, cells were treated with supernatant of cartilage lyste or poly (I:C) for RNA or protein collection.

### Statistical analysis

All data representative of three independent experiments are present as mean±S.E.M. We used two-tailed *t-*tests to determine significances between two groups. We did analyses of multiple groups by one- or two-way ANOVA with Bonferroni post-test of GraphPad prism version 5. For all statistical tests, we considered *P*-value <0.05 to be statistically significant.
