# Supplementary material for: Double-stranded RNA released from damaged articular chondrocytes promotes cartilage degeneration via Toll-like receptor 3-interleukin-33 pathway
Source: Cell Death Dis. 2017 Nov 2;8(11):e3165–. doi: 10.1038/cddis.2017.534 (PMC5775407; doi:10.1038/cddis.2017.534)
Supplement: Supplementary Figures [file cddis2017534x1.docx]

**Double-stranded RNA released from damaged articular chondrocytes promotes cartilage degeneration via Toll-like receptor 3–interleukin-33 pathway**

**Changwei Li^1#^, Kaizhe Chen^1,2#^, Hui Kang^1,3#^, Yufei Yan^1,2^, Kewei Liu^1^, Changjun Guo^1^, Jin Qi^1^, Kai Yang^1^, Fei Wang^1^, Lei Guo^1^*, Chuan He^1,2^*, Lianfu Deng^1^***

^1^ Shanghai Key Laboratory for Prevention and Treatment of Bone and Joint Diseases with Integrated Chinese-Western Medicine, Shanghai Institute of Traumatology and Orthopedics, Ruijin Hospital, Shanghai Jiaotong University School of Medicine, 197 Ruijin 2nd Road, Shanghai, 200025, People's Republic of China.

^2^ Department of Orthopedics, Ruijin Hospital, Shanghai Jiaotong University School of Medicine, 197 Ruijin 2nd Road, Shanghai, 200025, People's Republic of China.

^3^ Department of Orthopedics, Shanghai Tenth People's Hospital, Tongji University School of Medicine, Shanghai 200072, People's Republic of China.

**^#^** **These authors contributed equally to this work**

* **Corresponding author:**

Lianfu Deng

Shanghai Key Laboratory for the Prevention and Treatment of Bone and Joint Diseases with Integrated Chinese-Western Medicine, Shanghai Institute of Traumatology and Orthopedics, Rui Jin Hospital, Shanghai Jiao Tong University School of Medicine. Address: No. 197, Ruijin 2nd Road, Shanghai 200025 China; Zip Code: 200025; Fax: +86 21 64335742；Tel.: +86 21 64313534; E-mail: [lf_deng@126.com](mailto:lf_deng@126.com)

Lei Guo

Shanghai Key Laboratory for the Prevention and Treatment of Bone and Joint Diseases with Integrated Chinese-Western Medicine, Shanghai Institute of Traumatology and Orthopedics, Rui Jin Hospital, Shanghai Jiao Tong University School of Medicine. Address: No. 197, Ruijin 2nd Road, Shanghai 200025 China; Zip Code: 200025; Tel.: +86 21 64313534; E-mail: [guolei607@126.com](mailto:guolei607@126.com)

Chuan He

Shanghai Key Laboratory for the Prevention and Treatment of Bone and Joint Diseases with Integrated Chinese-Western Medicine, Shanghai Institute of Traumatology and Orthopedics, Rui Jin Hospital, Shanghai Jiao Tong University School of Medicine. Address: No. 197, Ruijin 2nd Road, Shanghai 200025 China; Zip Code: 200025; Tel.: +86 21 64313534; E-mail: [drhechuan@sina.com](mailto:drhechuan@sina.com)

**Supplementary figures**

**
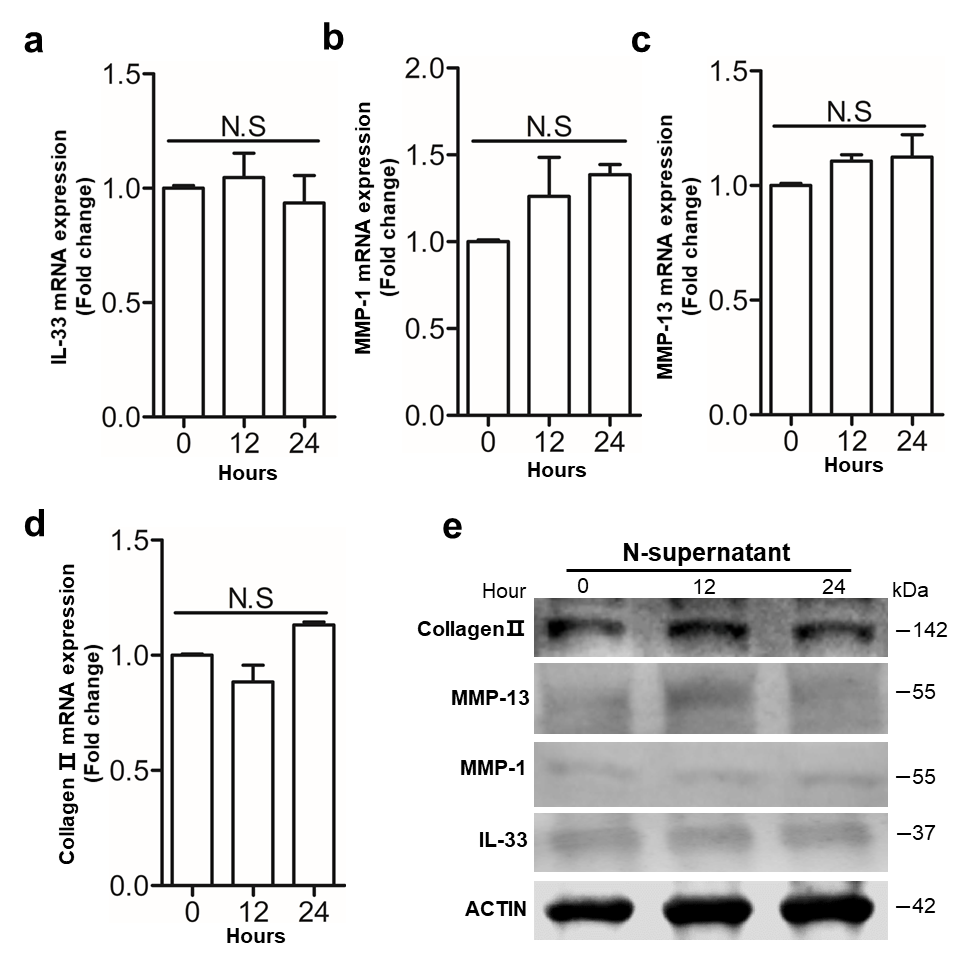
**

**Supplementary figure 1. IL-33, MMP-1, MMP-13 and collagen II expression in human chondrocytes induced by the** **supernatant of healthy cartilage lysate.** (a-d) Quantification of *Il-33* (a), *Mmp-1* (b), *Mmp-13* (c) and *Collagen* *II* (d) expression in human chondrocytes induced by supernatant of healthy cartilage lysate. (e) Western blot of IL-33, MMP-1, MMP-13 and collagen II in in human chondrocytes induced by supernatant of healthy cartilage lysate. N-supernatant represents supernatant from normal cartilage lysate. N.S means no significant difference, that is **P>0.05*. *P*-values were analyzed by one-way ANOVA.


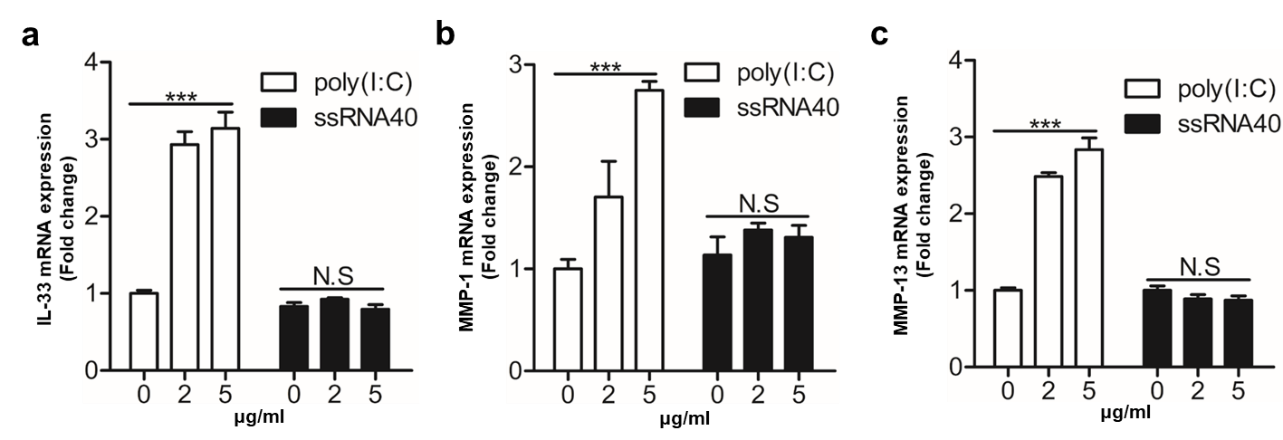


**Supplementary figure 2. *Il-33*, *Mmp-1* and *Mmp-13* expression in human chondrocytes induced by poly(I:C) and ssRNA40.** (a-c) Quantification of *Il-33* (a), *Mmp-1* (b), and *Mmp-13* (c) expression in human chondrocytes induced different doses of poly(I:C) and ssRNA40 for 24 hours. N.S means no significant difference, that is **P>0.05*. ****P<0.001*. *P*-values were analyzed by one-way ANOVA.
